# Coronary Vasospasm, an Unexpected Side Effect of Adenosine: A Case Report and Review of the Literature

**DOI:** 10.1155/cric/9174976

**Published:** 2025-11-14

**Authors:** Nadeem Jimidar, Tijs Bringmans, Johan Saenen, Emeline Van Craenenbroeck

**Affiliations:** Department of Cardiology, Antwerp University Hospital, Edegem, Belgium

**Keywords:** adenosine, case report, coronary spasm, microvascular, QT

## Abstract

Adenosine is a well-known product in the field of cardiology, known to cause coronary vasodilation. However, in rare cases, it has been associated with coronary vasoconstriction. In this case—the first to our knowledge—we have demonstrated that adenosine could be the cause of microvascular coronary spasms, based on the symptoms, ECG, and coronary functional assessments. Additionally, we have linked this case with a transient prolonged QT and mild dilated left ventricle, which was further investigated with a loop recorder and genetic testing revealing variants in the LMNA gene and PRKAG2 gene. This case also emphasizes that physicians should always take into account unexpected side effects during standard procedures.

## 1. Introduction

Adenosine, an endogenous purine nucleoside, is a well-known product in the field of cardiology. Its cardiac receptors are located in the coronary arteries, sinoatrial node, atrial myocytes, and atrioventricular (AV) node [1, 2]. Adenosine is a known vasodilator, and it is the first-line pharmacological treatment option for acute AV node-dependent tachycardias and can be used for the differential diagnosis of wide-QRS tachycardia. In the cathlab, adenosine is routinely used to induce coronary hyperaemia, either by intracoronary (IC) bolus injection or through intravenous (IV) infusion [2, 3]. While the most prevalent side effect is asthmatic bronchospasm, severe pro-arrhythmogenic responses have been reported, such as torsades des pointes, sustained ventricular tachycardia (VT) and ventricular fibrillation (VF) [4–6]. In the past, only a few reports have suggested the existence of possible adenosine-induced vasoconstriction. In this case, we demonstrate the specific possibility of adenosine-induced microvascular coronary spasms.

## 2. Case Presentation

A 47-year-old female presented with fatigue, episodes of palpitations, and postural dizziness. Prior medical history was significant for paroxysmal palpitations, with documentation of six beats of nonsustained VT and mild mitral valve regurgitation. No hereditary cardiac conditions were suspected based on her family history.

**Figure 1:**
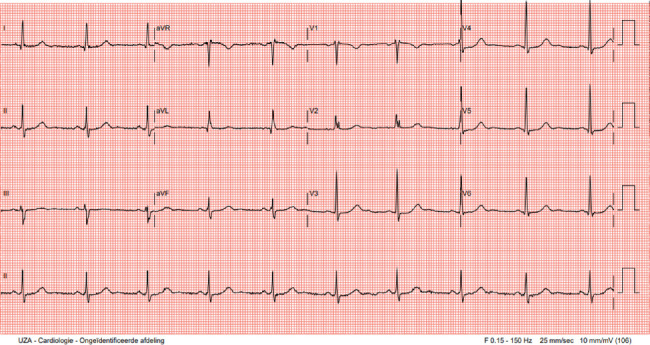
ECG in rest: sinus rhythm 60 bpm, rather short PR < 150 ms, QRS 100 ms, normal axis, RSR' in V1, QTc 480 ms.

Physical examination was unremarkable. Resting electrocardiogram (ECG) showed sinus rhythm at 60 bpm, a PR interval of 145 ms with possible ventricular pre-excitation in Leads II and aVF, an rSr' pattern in V1, and a measured QTc according to Bazett's method of 480 ms (Figure 1).

A bicycle stress test did not show ischemic repolarization abnormalities nor ventricular ectopy, and the pre-excitation pattern in the inferior leads did not change during exercise.

**Figure 2:**
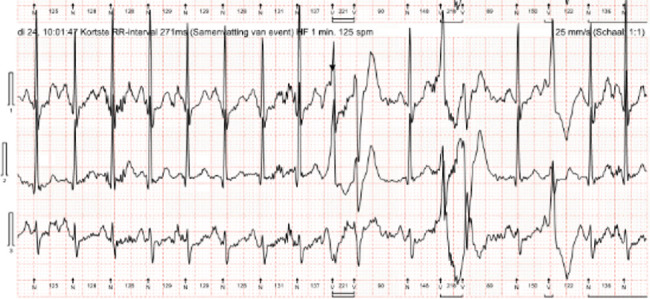
Polymorphic PVC couplets during 1-day ECG monitoring.

One-day heart rhythm monitoring disclosed sinus rhythm with a low burden (< 1%) of premature ventricular contractions (PVCs); however, several polymorphic couplets occurred (Figure 2). Individualized QT interval (QTi) measured 439 ms, with intermittent QTc prolongation to a maximal 490 ms.

**Figure 3:**
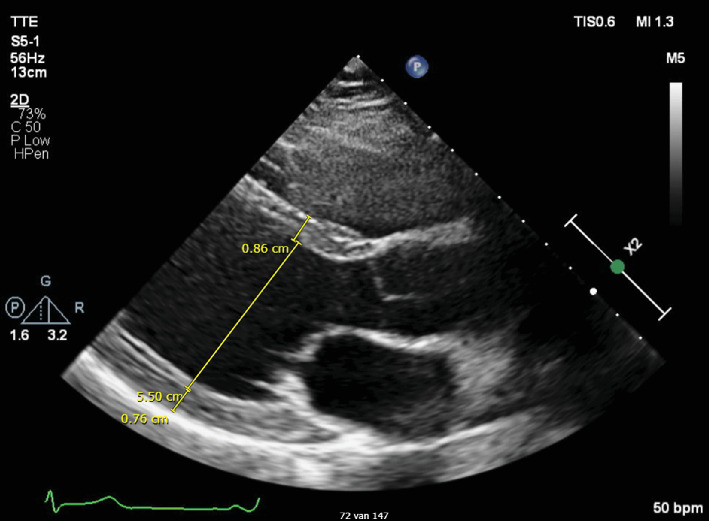
Echocardiography showing a mild dilated, nonhypertrophic ventricle.

Echocardiography showed a nonhypertrophic (end-diastolic interventricular septum thickness 8.6 mm, posterior wall thickness 7.6 mm), mild dilated left ventricle (left ventricular end-diastolic diameter 55 mm) with preserved ejection fraction, and billowing of both mitral valve leaflets with mild regurgitation (Figure 3).

Cardiac magnetic resonance scan confirmed mild dilation of the left ventricle (indexed left ventricular end-diastolic volume 120 ml/m^2^), normal systolic function (left ventricular ejection fraction 57%), and mild mitral regurgitation (regurgitation fraction 17%). A small zone of patchy fibrosis at the basal anterior and basal inferior sides of the left ventricle was noted.

**Figure 4:**
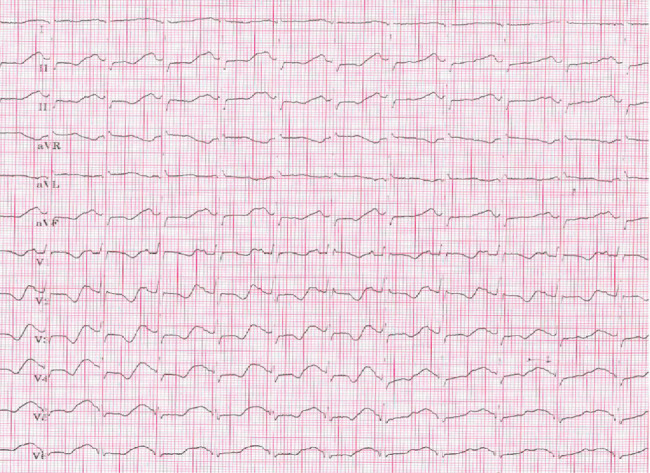
Diffuse ischemic ECG changes after 6 mg adenosine IV.

As the patient experienced palpitations and the resting ECG was suspected for a possible pre-excitation syndrome, an adenosine drug test was performed to unmask accessory pathway conduction. After an IV bolus of 6 mg adenosine, the patient developed severe anginal chest pain with diffuse horizontal ST segment depressions, T wave inversion, and ST segment elevation in aVR, suggestive of diffuse myocardial ischemia (Figure 4). QT/QTc increased to 480/620 ms with Bazett's formula. After 10 s, the chest pain dissolved and the ECG normalized. Similar observations occurred after 12 mg of adenosine IV. Manifest pre-excitation and complete AV block were not observed; therefore, the adenosine test was deemed inconclusive.

**Figure 5:**
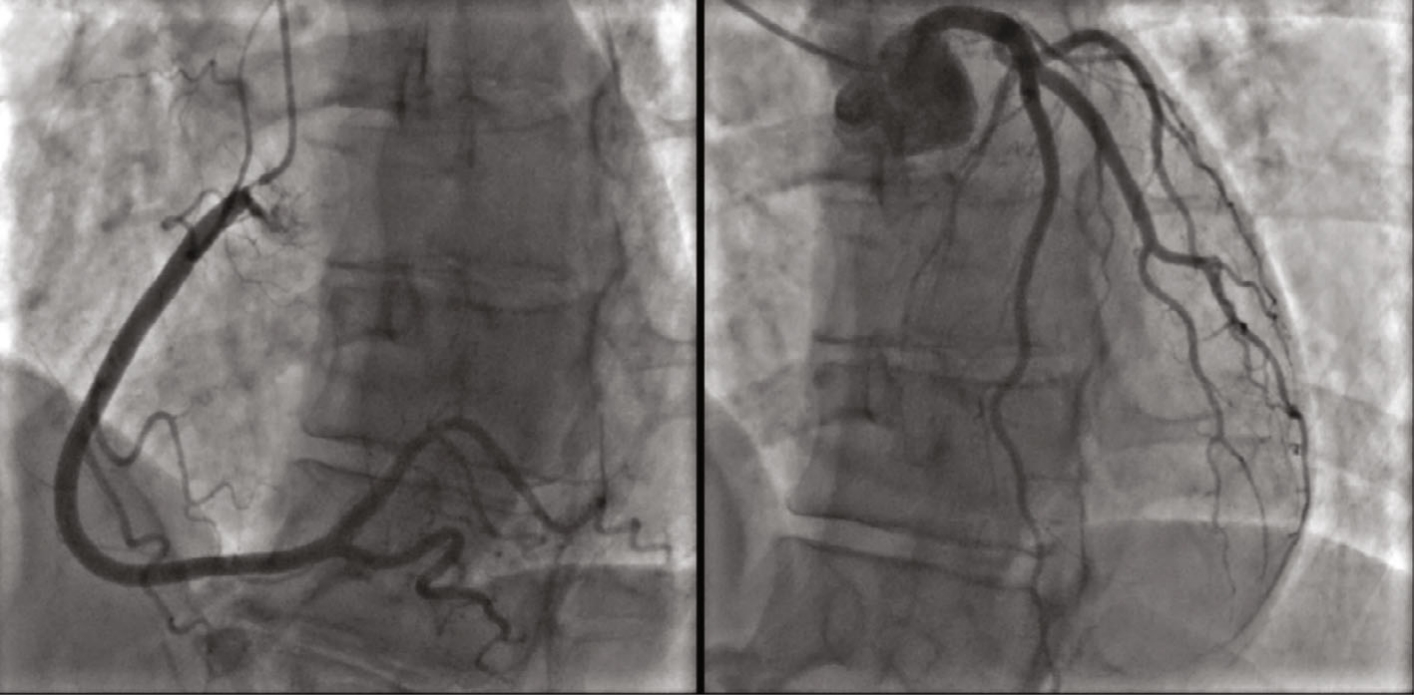
Coronary angiography showed normal coronary arteries, no epicardial spasm after 12 mg adenosine IV.

**Figure 6:**
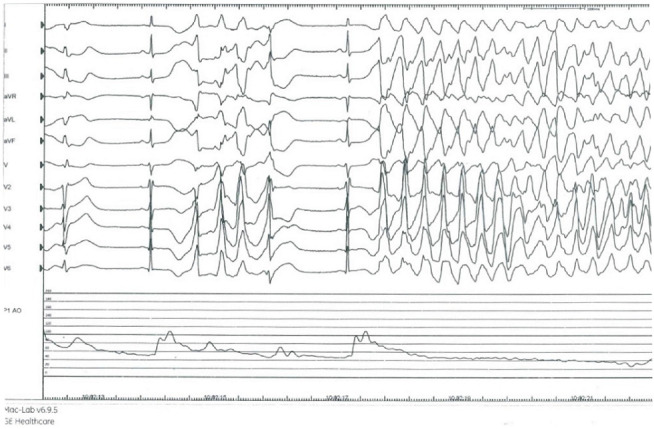
Significant QT prolongation after 12 mg papaverine IC with early afterdepolarization and resultant polymorphic VT with degeneration into ventricular fibrillation.

Because of the phenomenon of adenosine-induced chest pain and ischemic abnormalities, the patient was referred for coronary angiography at our center, which showed normal coronary arteries (Videos S1–S3). Subsequently, IV bolus adenosine 12 mg was given, which replicated prior clinical and electrocardiographic ischemic responses, without epicardial spasm (Figure 5). Next, coronary physiology was performed with a pressure and temperature sensor equipped coronary wire (PressureWire X, Abbott) in the left anterior descending (LAD) artery. Resting full-cycle ratio was 0.96. Bolus thermodilution was subsequently performed to measure the coronary flow reserve and microvascular resistance. However, after IC injection of 12 mg papaverine, QT prolonged to 560 ms with short-coupled PVC that resulted in unstable VT which degenerated into VF (Figure 6). Electric defibrillation was performed successfully.

**Figure 7:**
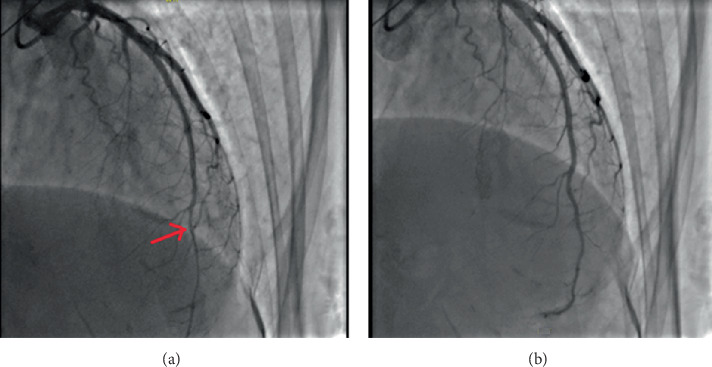
(a) Mild coronary artery vasoconstriction in the distal left anterior descending after 200 *μ*g acetylcholine. (b) Reference coronary artery diameter after isosorbide dinitrate IC.

**Figure 8:**
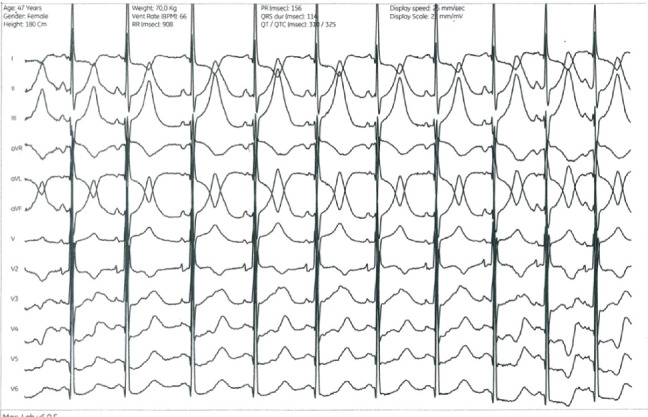
ECG after acetylcholine administration. Notice the diffuse ST depression with different T morphology compared with Figure 3. Caveat 25 mm/s, 25 mm/mV.

After recovery of sinus rhythm, coronary vasoreactivity tests were performed with a step-up dosing protocol of IC acetylcholine (ACH). At the maximal dose of 200 *μ*g ACH, diffuse vasoconstriction occurred at the distal LAD, combined with subjectively different chest pain with radiation to the left shoulder, which she had never felt before (Figure 7). Vasoconstriction and chest discomfort resolved after IC nitrates. During the ACH test, diffuse ST depression occurred with different T morphology compared with the adenosine test (Figure 8).

Further follow-up included a genetic panel for channelopathies and continuous rhythm monitoring with an internal loop recorder. After 36 months, no ventricular arrhythmia was recorded, other than an intermittent high burden of PVC (up to 13%/24 h).

Her deoxyribonucleic acid (DNA) analysis revealed a variant in the LMNA gene at the level of Exon 5 (heterozygote C.884C > T, p.(Ser295Leu) and a variant in the PRKAG2 gene at c.1475T > A p.(Ile492Asn). Furthermore, at the level of the GNB5 gene, there was a heterozygote c.62del p.(Arg21Glnfs∗3) pathogenic variant (Class 5/5 pathogenicity).

## 3. Discussion

We present a case of a 47-year-old woman with palpitations who had a prolonged QTc interval with possible ventricular pre-excitation syndrome. As QTi was 439 ms, she was not considered as having long QT syndrome (LQTS) [7]. During the adenosine IV study, she developed severe chest discomfort with diffuse ischemic ECG changes and marked QTc prolongation to 620 ms. Because of chest pain symptoms and ischemic ECG changes, we also suspected adenosine-induced coronary artery spasm and referred the patient for invasive coronary function tests.

Adenosine usually induces coronary vasodilation and thus increased coronary blood flow through A2 receptor signalling and exerts its electrophysiological effects through A1 receptors. The A1 receptors couple with G-protein-coupled, inward-rectifying potassium (GIRK) channels, similar to the mechanism of the muscarinic ACH receptor. GIRK channels are responsible for the inwardly rectifying potassium (IKAdo) current, which causes membrane hyperpolarization. In addition, the Galphai subunit of the A1 receptor inhibits cyclic adenosine monophosphate (cAMP) production, therefore reducing the beta1-adrenoreceptor response to catecholamines and reducing L-type Ca2+ channel currents, similar to the muscarinic pathways. Because of a greater receptor reserve and therefore downstream amplifying signalling pathways, adenosine is 11 times more efficacious at inhibiting beta 1-adrenoreceptor response than activating IKAdo [8].

In contrast, a few case reports of suspected adenosine-induced coronary vasospasm have been reported in the literature [9–12]. In 2015, a Korean publication claimed to be the first to detect coronary artery spasms after adenosine was used during a computed tomography scan [13].

Coronary angiographic proof was demonstrated in 2022 [14], when a patient was given adenosine for checking dormant conduction in the pulmonary veins at the end of a pulmonary vein isolation procedure. Shortly after, the ECG showed ST segment elevation, then left bundle branch block, followed by AV block, all combined with hemodynamic deterioration. Ad hoc coronary angiography showed diffuse coronary artery spasm, treated with nitroglycerin.

The underlying mechanism of adenosine provoked coronary vasoconstriction is not well understood. One hypothesis is based on the presence of *A1* adenosine receptors in the coronary arterioles. As we know from the afferent arterioles of the nephron, stimulation of A1 receptors by exogenous adenosine causes smooth vascular contraction [13, 15, 16]. This was also demonstrated in an A1-adenosine receptor knockout model: stimulating A1 receptors resulted in vasoconstriction involving phospholipase C pathways [17].

Others suggested that the short half-time of adenosine levels in our system—and thus short maximum vasodilation state—followed by rapid decline in levels, could result in a period of “relative” vasoconstrictive state at the coronary artery level [9, 18]. A third hypothesis links the problem not with the adenosine receptor, but with the adenosine triphosphate-sensitive potassium channel. After all, adenosine also directly stimulates these channels, regulating vascular tone. Structural changes in these channels could lead to altered reactions such as spasms instead of dilation [19].

In our patient, the indication for adenosine administration consisted of the exclusion of a Kent bundle, as ventricular pre-excitation was considered possible [20]. To our knowledge, this is the first case of suspected coronary vasospasm in which additional coronary function tests were performed.

The most important findings here are that adenosine IV did not cause *epicardial* coronary artery spasm, despite the ischemic ECG changes seen. This contradicts the observation seen by Ahmed et al. [14], but the chest pain, ST depression, and the beneficial effect of nitrates may suggest that adenosine can cause *isolated microvascular* spasm [21, 22].

In addition, the ACH vasoreactivity test was not diagnostic for epicardial coronary artery spasm conforming to the Coronary Vasomotor Disorders International Study (COVADIS) group consensus document, as no ≥ 90% spasm occurred, and as the patient did not recognize the provoked chest discomfort [21]. These findings suggest that the mechanism of ischemia during adenosine IV is not caused by stimulation of the IKAdo current, as muscarinic stimulation with ACH is believed to stimulate the same GIRK channels.

Apart from the microvascular vasoconstriction due to adenosine, significant QTc prolongation was observed with both adenosine IV and papaverine IC. QT prolongation after adenosine may be caused by both ischemia [23] due to microvascular spasm, as well as by an intrinsic effect of adenosine itself [8]. QT prolongation after papaverine is a known effect, and ventricular arrhythmia after bolus injection of papaverine has been described [24, 25]. Given the patient's presentation—including a mildly dilated left ventricle, polymorphic PVC couplets on Holter monitoring, prior documentation of short nonsustained VT, an unexplained response to adenosine IV, and VF due to drug-induced QT prolongation—additional genetic tests were performed in combination with the implantation of a loop recorder.

Three years later, no sustained ventricular arrhythmias had occurred, except for intermittent high burden of PVC. Genetic tests revealed heterozygote variants in the LMNA gene and PRKAG2 gene, the pathogenicity of which is at this moment still of uncertain significance. The LMNA variant has been documented in hypertrophic cardiomyopathy [26], dilated cardiomyopathy, and long QT syndrome. The PRKAG2 variant has been reported in a case with hypertrophic cardiomyopathy [27]. As the pathogenic variant in the GNB5 gene is recessive in nature, the heterozygote state is not considered of pathogenic significance.

In conclusion, we present the case of a patient with a prolonged QT interval and mildly dilated left ventricle who underwent an adenosine drug test to unmask ventricular pre-excitation. During the administration of adenosine, the patient exhibited diffuse repolarization disturbances with chest pain. Based on our investigations, we excluded epicardial vasospasm and suspect microvascular spasm, provoked by adenosine. To the best of our knowledge, this is the first report of this adenosine-induced microvascular vasoconstriction.

## Data Availability

The data that support the findings of this study are available on request from the corresponding author. The data are not publicly available due to privacy or ethical restrictions.

## Supporting Information

Supporting Information Additional supporting information can be found online in the Supporting Information section. Video S1 and S2: Coronary angiograms of the right coronary artery. Video S3: Coronary angiograms of the left coronary artery in baseline setting, at the start of the procedure.

## References

[1] Wilbur S. L., Marchlinski F. E. (1997). Adenosine as an Antiarrhythmic Agent. *American Journal of Cardiology*.

[2] Belardinelli L., Linden J., Berne R. M. (1989). The Cardiac Effects of Adenosine. *Progress in Cardiovascular Diseases*.

[3] Camm A. J., Lüscher T. F., Maurer G., Serruys P. W. (2018). *The ESC Textbook of Cardiovascular Medicine*.

[4] Harrington G. R., Froelich E. G. (1993). Adenosine-Induced Torsades de Pointes. *Chest*.

[5] Huemer M., Boldt L. H., Rolf S., Blaschke D., Parwani A., Haverkamp W. (2009). Sustained Monomorphic Ventricular Tachycardia After Adenosine Infusion. *International Journal of Cardiology*.

[6] Kaplan I. V., Kaplan A. V., Fisher J. D. (2000). Adenosine Induced Atrial Fibrillation Precipitating Polymorphic Ventricular Tachycardia. *Pacing and Clinical Electrophysiology*.

[7] Robyns T., Nuyens D., Vandenberk B. (2023). Individualized QT Interval (QTi) Is a Powerful Diagnostic Tool in Long QT Syndrome: Results From a Large Validation Study. *Frontiers in Cardiovascular Medicine*.

[8] Matthews G. D., Grace A. A. (2020). Unmasking Adenosine: The Purinergic Signalling Molecule Critical to Arrhythmia Pathophysiology and Management. *Arrhythmia & Electrophysiology Review*.

[9] Arora P., Bhatia V., Arora M., Kaul U. (2014). Adenosine Induced Coronary Spasm - a Rare Presentation. *Indian Heart Journal*.

[10] Polad J. E., Wilson L. M. (2002). Myocardial Infarction During Adenosine Stress Test. *Heart*.

[11] van der Hiel B., Scholte A. J., Stokkel M. P. (2007). Intermittent ST-Segment Depressions During Adenosine Stress Test. *Clinical Nuclear Medicine*.

[12] Weissman G., Scandrett R. M., Howes C. J., Russell R. R. (2004). Coronary Vasospasm During an Adenosine Stress Test. *Journal of Nuclear Cardiology*.

[13] Nam J. G., Choi S. H., Kang B. S., Bang M. S., Kwon W. J. (2015). Development of Coronary Vasospasm During Adenosine-Stress Myocardial Perfusion CT Imaging. *Korean Journal of Radiology*.

[14] Ahmed J. J., Walborn D. L., Anghel T. M., Chohan M. R. (2022). Acute STEMI Due to Severe Triple-Vessel Spasm After IV Adenosine Injection During Cryo-Balloon Isolation. *JACC: Case Reports*.

[15] Vallon V., Osswald H. (2009). Adenosine Receptors and the Kidney. *Handbook of Experimental Pharmacology*.

[16] Sato A., Terata K., Miura H. (2005). Mechanism of Vasodilation to Adenosine in Coronary Arterioles From Patients With Heart Disease. *American Journal of Physiology. Heart and Circulatory Physiology*.

[17] Tawfik H. E., Schnermann J., Oldenburg P. J., Mustafa S. J. (2005). Role of A1adenosine Receptors in Regulation of Vascular Tone. *American Journal of Physiology. Heart and Circulatory Physiology*.

[18] Rosenberg T., Perdrisot R. (2008). Coronary Spasm After an Adenosine Stress Test: An Adverse Effect of a Vasodilator. *Acta Cardiologica*.

[19] Quevedo H. C., Munoz-Mendoza J., Pinto Miranda V., Sequeira R. F. (2013). Coronary Vasospasm while Treating Supraventricular Tachycardia: Is Adenosine Really to Blame?. *Case Reports in Cardiology*.

[20] Belhassen B., Fish R., Viskin S., Glick A., Glikson M., Eldar M. (2000). Adenosine-5’-Triphosphate Test for the Noninvasive Diagnosis of Concealed Accessory Pathway. *Journal of the American College of Cardiology*.

[21] Beltrame J. F., Crea F., Kaski J. C. (2017). International Standardization of Diagnostic Criteria for Vasospastic Angina. *European Heart Journal*.

[22] Saraste A., Barbato E., Capodanno D. (2019). Imaging in ESC Clinical Guidelines: Chronic Coronary Syndromes. *European Heart Journal Cardiovascular Imaging*.

[23] Kenigsberg D. N., Khanal S., Kowalski M., Krishnan S. C. (2007). Prolongation of the QTc Interval Is Seen Uniformly During Early Transmural Ischemia. *Journal of the American College of Cardiology*.

[24] Okabe Y., Otowa K., Mitamura Y. (2018). Evaluation of the Risk Factors for Ventricular Arrhythmias Secondary to QT Prolongation Induced by Papaverine Injection During Coronary Flow Reserve Studies Using a 4 Fr Angio-Catheter. *Heart and Vessels*.

[25] Nakayama M., Tanaka N., Sakoda K. (2015). Papaverine-Induced Polymorphic Ventricular Tachycardia During Coronary Flow Reserve Study of Patients With Moderate Coronary Artery Disease. *Circulation Journal*.

[26] Lopes L. R., Syrris P., Guttmann O. P. (2015). Novel Genotype-Phenotype Associations Demonstrated by High-Throughput Sequencing in Patients With Hypertrophic Cardiomyopathy. *Heart*.

[27] Walsh R., Thomson K. L., Ware J. S. (2017). Reassessment of Mendelian Gene Pathogenicity Using 7,855 Cardiomyopathy Cases and 60,706 Reference Samples. *Genetics in Medicine*.

